# Socioeconomic and Clinical Risk Factors for Meconium-Stained Amniotic Fluid and Associated Maternal–Neonatal Morbidity in Ethiopia: A Prospective Case–Control Study

**DOI:** 10.3390/ijerph23020231

**Published:** 2026-02-11

**Authors:** Loris Marin, Guido Ambrosini, Elisabetta Valentini, Jordyn Conley, Alessandra Andrisani

**Affiliations:** Department of Women’s and Children’s Health, University of Padua, Via Giustiniani 3, 35128 Padua, Italy

**Keywords:** meconium-stained amniotic fluid, health care, low-income countries, Ethiopia, pregnancy

## Abstract

Meconium-stained amniotic fluid (MSAF) results from premature release of meconium by the fetus under stressful conditions and is associated with increased risk of maternal and neonatal morbidity and mortality. Risk factors for stressful conditions may differ between women living in highly developed countries and those in low-income countries. This study aimed to evaluate known and potential risk factors for MSAF and to assess the association between MSAF and maternal and neonatal morbidity. This prospective case–control study was conducted at a tertiary care hospital in Wolisso, Ethiopia. A total of 165 women were enrolled and divided into two groups: group A (65 women with MSAF) and group B (100 women with clear amniotic fluid). Data were collected through medical records (pregnancy, maternal and fetal outcomes) and questionnaires (socioeconomic factors). Women with MSAF had statistically significant differences in distance traveled, means of transportation, travel time to reach the hospital, weekly workload, and family income compared to controls. Higher rates of intrapartum monitoring abnormalities and operative deliveries were also observed among women with MSAF. The socioeconomic situation of pregnant women referred to the hospital in Wolisso appears to be related to the occurrence of MSAF. Recognizing these risk factors is crucial to improving quality of care and maternal–fetal health.

## 1. Introduction

Meconium-stained amniotic fluid (MSAF) is recognized as a sign of fetal distress and it is associated with increased neonatal intensive care unit admissions, increased perinatal morbidity and mortality, maternal infections, and operative and cesarean deliveries [1,2]. Meconium emission in utero can be due to fetal sphincter release [3]. The occurrence of meconium-stained amniotic fluid (MSAF) ranges from 8 to 15% of deliveries [2] and it is known to be more frequent and more severe in areas with suboptimal control of pregnancies [3]. Known risk factors are post-term pregnancies, prolonged labor, clinical chorioamnionitis, fetal growth restriction, preeclampsia, oligohydramnios, vaginal breech delivery, maternal drugs (e.g., cocaine, castor oil, bowel purgatives), herbal substances (e.g., “isihlambezo”), and intrahepatic cholestasis of pregnancy [4]. Meconium aspiration syndrome (MAS) reflects a spectrum of disorders in infants born with meconium-stained amniotic fluid, and its symptoms range from mild tachypnea to severe respiratory distress and significant mortality [5].

In Ethiopia, nearly 80.5% of the total population lives in rural areas and Ethiopia’s economy is mainly based on rainfed and subsistence agriculture. Women in Africa are economically active, and they carry out most agricultural activities. African women work an average of 17 h a day, devoting themselves to daily activities that involve caring for the family and raising children. They also take care of daily activities such as preparing meals and obtaining water, which can take several hours since wells, fields and markets are often located at long distances from home and not accessible by means of transport [6].

The aim of this study is to evaluate whether stressful socioeconomic factors that are not present in high-income countries can be considered as risk factors for MSAF. Secondly, maternal and neonatal morbidity is also evaluated.

## 2. Materials and Methods

This is a prospective case–control study performed at a tertiary-care, private, nonprofit hospital in Ethiopia. Data was collected between 2023 and 2024 through the consultation of medical records and an interviewer-administered questionnaire.

The study was conducted in accordance with the principles of the Declaration of Helsinki and was approved by the Ethics Committee of St. Luke Catholic Hospital and College of Nursing and Midwifery, Wolisso, Ethiopia (reference number 1634A/2022, June 2022). The study was classified as a non-interventional observational project based exclusively on routinely collected clinical data, and no changes to standard clinical practice were introduced. All participants were informed about the study, and verbal informed consent for the anonymized use of their data for research purposes was obtained prior to inclusion. The data collected through the interviewer-administered questionnaire covered the following variables: travel time to get to the hospital, mode of travel, city and neighborhood of origin, number of weekly hours worked, monthly household income, number of living children, and antenatal care follow-up.

The inclusion criteria were full-term pregnant women. The exclusion criteria were known obstetric or fetal pathologies, cases in which MSAF arose after admission for delivery or during the second stage of labor, the use of herbal products and preparations, and patient refusal to participate in the study. Enrolled women were divided into two groups according to the presence of meconium-stained amniotic fluid (group A) and presence of clear amniotic fluid (group B).

Primary outcomes of the study were to evaluate known and potential risk factors for MSAF: labor duration, labor augmentation, premature rupture of membranes (PROM), diagnosis of oligohydramnios on admission, antenatal care follow-up, the distance traveled to the hospital, trip duration, the means of transport used, weekly workload, family income, and the number of children at home.

Secondary outcomes were evaluation of maternal and neonatal morbidity: temperature elevation during labor, labor dystocia, and modes of delivery were evaluated. Regarding newborns parameters, neonatal birth weight, APGAR score and need of resuscitation were evaluated.

### Statistical Analysis

Continuous numerical data were expressed as absolute and relative frequencies (%), and purely numerical data as mean ± standard deviation. Parametric and non-parametric tests were used. A *p*-value less than 0.05 was considered statistically significant.

## 3. Results

A total of 165 participants were enrolled in the study, 65 MSAF cases, 39.4% (group A), and 100 cases with clear amniotic fluid, 60.6% (group B). No significant differences were found regarding age and parity. The age of women in group A was 26.1 ± 5.1, and 26.5 ± 5.8 in group B; *p*-value 0.69. Multiparous women made up 54% of group A and 66% of group B; *p*-value 0.12 (Table 1). MSAF was found in 30 primiparous women (46.9%), while in multiparous patients MSAF was found in 34.6% of cases (35 patients); *p*-value 0.12. Among multiparous women, the interval between the previous and current pregnancy was 16.6 ± 9.1 months in group A and 15.9 ± 7.4 months in group B; *p*-value: 0.71.

Four admission diagnoses were noted: active first stage of labor (AFSOL), latent first stage of labor (LFSOL), second stage of labor, and rupture of membranes (ROM). The diagnosis at admission was AFSOL in 25 cases of group A (38.5%) and in 28 of group B (28%); LFSOL in 5 cases of group A (7.7%) and in 28 of group B (28%); second stage of labor in 9 cases of group A (13.8%) and in 25 of group B (25%); and ROM in 26 cases of group A (40%) and in 19 of group B (19%).

No significant differences were found between the two groups in the duration of labor of enrolled women, diagnosis of oligohydramnios on admission, diagnosis of PROM and augmentation during labor between group A and group B (Table 1).

Labor duration of women in group A was 9.1 ± 3.9 h and 9.1 ± 4.9 in group B; *p*-value 1. Augmentation during labor was implemented in 10 (15.4%) patients in group A and in 15 (15%) in group B; *p*-value 0.94. Oligohydramnios was detected in only one case in group A and in three cases in group B. Premature rupture of membranes (PROM) was detected in 6 out of 65 women of group A (9.2%) and in 11 out of 100 women of group B (11%); *p*-value 0.71.

**Figure 1:**
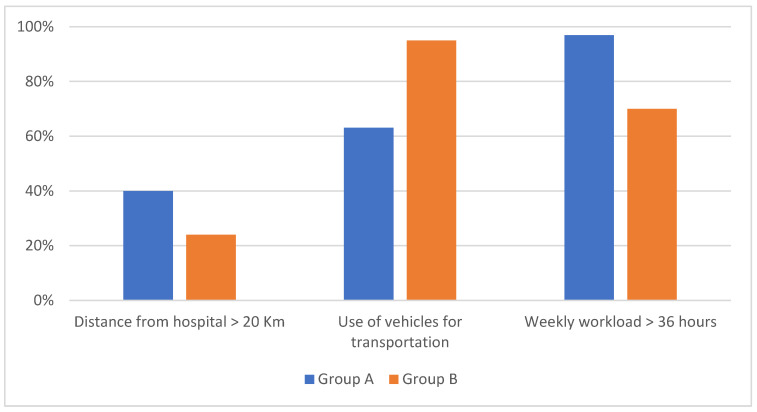
Representation of socioeconomic risk factors for meconium-stained amniotic fluid (MSAF) in group A (women with meconium-stained amniotic fluid) and group B (women with clear amniotic fluid).

In terms of socioeconomic status, 98 women had received an antenatal care follow-up (59.4%): 37 women in group A (56.9%) and 61 women in group B (61%); *p*-value 0.6. The distance traveled to St. Luke’s Hospital was assessed. In the study, the distance was 33.1 ± 54 km in group A and 18.6 ± 27.2 in group B; *p*-value 0.02. A total of 50 women traveled 20 km or more: 26 women in group A (40%) and 24 women in group B (24%); *p*-value 0.03 (Figure 1).

**Figure 2:**
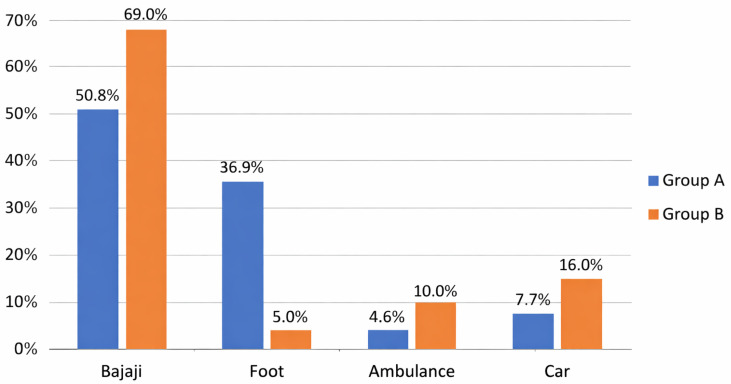
Representation of transport modes used by the sample, with comparison between group A (women with meconium-stained amniotic fluid) and group B (women with clear amniotic fluid).

Means of transport were also taken into account: by bajaji (a typical motorized mean of transport), on foot, by ambulance, or by car. Overall, 102 women traveled by bajaji (61.8%), 29 on foot (17.6%), 13 by ambulance (7.9%), and 21 by car (12.7%). Among the women in group A, 33 traveled by bajaji (50.8%), 24 on foot (36.9%), 3 by ambulance (4.6%), and 5 by car (7.7%). Among the women in group B, 69 (69%) traveled by bajaji, 5 (5%) on foot, 10 (10%) by ambulance, and 16 (16%) by car. A total of 41 women traveled by different means of transport in group A (63.1%) and 95 women in group B (95%); *p*-value < 0.001 (Figure 2).

Trip duration to reach the hospital was significantly higher in group A compared to group B. In fact, it took 65.5 ± 75.5 min in group A and 26 ± 33.2 min in group B; *p*-value < 0.001 (Table 1). The weekly workload and family income were also analyzed. A total of 133 women claimed to work more than 36 h per week (80.6%): among these patients, 63 women were in group A (96.9%), while 70 women were in group B (70%); *p*-value < 0.001 (Figure 1). Regarding family income, the monthly income was 2149.23 ± 743.54 birr in group A and 2655 ± 571.61 birr in group B; *p*-value < 0.001. The number of children at home was 1.4 ± 1.8 in group A and 1.7 ± 1.9 in group B; *p*-value = 0.34 (Table 1).

**Figure 3:**
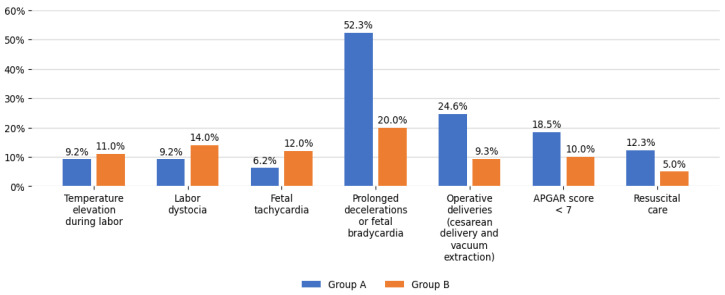
Representation of maternal and neonatal morbidity in group A (women with meconium-stained amniotic fluid) and group B (women with clear amniotic fluid).

Regarding maternal morbidity, temperature elevation during labor, labor dystocia, intrapartum fetal monitoring, and modes of delivery were evaluated. Elevated body temperature (higher than 37.5 °C) was detected in 17 women: 6 in group A and 11 in group B; *p*-value 0.71 (Figure 3).

Labor dystocia occurred in 20 patients in the study, of which 6 were among MSAF cases (9.2%) and 14 were among controls (14%); *p*-value 0.36 (Table 2).

During intrapartum fetal monitoring, alterations of fetal heart rate tracings were measured. Fetal tachycardia was detected in 4 cases in group A (6.2%) and 12 cases in group B (12%); *p*-value 0.22. Prolonged decelerations and/or fetal bradycardia emerged in 34 cases in group A (52.3%) and in 20 women in group B (20%); *p*-value < 0.001 (Figure 3). There were three modes of delivery identified in the sample: spontaneous vaginal delivery, cesarean section and operative delivery by vacuum extraction (Table 2) (Figure 3). Three babies had a breech presentation, all of which occurred in group B; two were born by cesarean section. Excluding breech presentations, in group A, 49 women (75.4%) had spontaneous delivery and 16 (24.6%) had operative delivery, including 12 (18.5%) by cesarean section and 4 (6.1%) by vacuum extraction (Table 2). In group B, 88 of 97 women (90.7%) had spontaneous delivery and 9 of 97 (9.3%) had op-erative delivery, including 8 of 97 (8.3%) by cesarean section and 1 of 97 (1.0%) by vacuum extraction. Operative deliveries were statistically higher in group A; *p*-value = 0.008 (Table 2) (Figure 3).

Regarding newborn parameters, neonatal birth weight, APGAR score and resuscitation care were evaluated. Neonatal birth weight in group A was 3043 ± 401 g and 3040 ± 567 g in group B; *p*-value 0.97. Neonatal birth weight was less than 2500 g in 16 women: 3 in group A (4.6%) and 13 in group B (13%); *p*-value 0.08. APGAR score was evaluated at the first minute of life of newborns. APGAR score < 7 was detected in 22 women: 12 in group A (18.5%) and 10 in group B (10%); *p*-value 0.12. A total of 13 newborns were resuscitated (7.9%): 8 in group A (12.3%) and 5 in group B (5%); *p*-value 0.09 (Table 2) (Figure 3).

## 4. Discussion

Many studies associate the occurrence of MSAF with various feto-maternal risk factors, resulting in a significantly increased risk of perinatal morbidity and mortality and feto-maternal infections [2]. Preventing serious complications may be achieved through early identification and management of these risks. This study examined MSAF in women attending a tertiary care hospital in Ethiopia, with attention to both clinical and socioeconomic conditions, in order to translate the widely recognized risk factors from Western contexts to the Ethiopian setting and to develop an individualized care plan.

Classical risk factors linked to MSAF include labor duration, augmentation, oligohydramnios, PROM, maternal fever, and labor dystocia [7]. In addition, this study considered broader socioeconomic variables such as workload, distance to hospital, travel time, type of transport, antenatal care, monthly family income and number of living children. These aspects are rarely addressed in high-income settings but may play a critical role in low-resource environments. In contrast with data reported in the literature, no associations were observed between MSAF and duration of labor, oligohydramnios, PROM, or augmentation [8,9,10]. This discrepancy may reflect the shorter average labor recorded in both groups in our sample compared to those reported elsewhere [8,9,10], as well as the relatively small number of cases diagnosed with oligohydramnios and PROM. Antenatal care follow-up showed no protective effect, suggesting that in low-income settings, socioeconomic stressors may also contribute to the risk of MSAF.

Analysis of these socioeconomic determinants revealed marked inequalities. Women with MSAF traveled significantly longer distances, often exceeding 20 km, and a greater proportion reached hospital on foot. As a result, their trip duration was longer and more physically demanding. Such conditions, especially when contractions had begun or after PROM, may generate maternal stress that triggers fetal sphincter relaxation and meconium passage [11]. Family income was lower in the MSAF group, and these women also reported heavier workloads. In rural Ethiopia, women perform agricultural tasks while also caring for children, cooking, and collecting water, which require hours of daily effort. These cumulative stressors likely contribute to adverse pregnancy outcomes [12]. Evidence from other contexts shows that moderate-intensity activity for 30–40 min per day is safe in pregnancy, associated with improved fetal tolerance to labor, reduced maternal weight gain, lower incidence of gestational diabetes, and even reduced risk of preeclampsia [13]. However, recommendations do not extend to high-intensity or prolonged daily exertion. Beyond 40 min, maternal glucose may decline and potential fetal consequences are uncertain [14,15]. Unlike trained athletes, who undertake supervised exercise programs tailored to gestational age, hydration, and maternal health, rural Ethiopian women face unregulated, physically demanding tasks that may expose both mother and fetus to dehydration, hypoglycemia, and increased lactate. This contextual difference highlights the importance of interpreting physical activity data with cultural sensitivity.

The correlation between socioeconomic stressors and MSAF underscores the need for education of the Ethiopian population. Pregnant women should be informed about the risks associated with intense workloads, and public health initiatives should encourage community support during pregnancy. Furthermore, logistic interventions are critical. Women who experience labor onset or PROM far from medical facilities should be offered ambulance transport to avoid long walks that may worsen fetal compromise.

Similar challenges in obstetric accessibility have been addressed in other African countries through community-based emergency transport interventions aimed at reducing delays in reaching care. For instance, in northern Uganda, a hospital-supported motorcycle transport system for laboring women significantly reduced referral delays and improved timely access to delivery facilities [16]. In Tanzania, the m-Mama program implemented in the Shinyanga region introduced community-managed taxis and motorcycle ambulances, achieving high referral volumes and demonstrating improved cost-effectiveness compared with standard ambulance services [17,18]. In Malawi, the use of motorcycle ambulances in rural areas reduced referral delays by 35–76% and decreased transport costs by up to 24-fold compared with car ambulances [19], while community bicycle ambulance and transport plan initiatives in southern Malawi strengthened obstetric referral systems [20]. A systematic review of transport interventions in low- and middle-income countries further highlights the potential of low-cost vehicles, community funds, and improved referral communication to reduce maternal and neonatal adverse outcomes [21].

Adapting such community-centered and low-cost transport models to the Ethiopian context through hospital–community collaboration, emergency transport funds and public education could represent a pragmatic and scalable approach to mitigating the socioeconomic and logistical barriers (distance, means of transport, travel time) identified in this study.

Prolonged decelerations or fetal bradycardia were registered during intrapartum fetal monitoring in women with MSAF, in accordance with data in the literature [22]. Consequently, operative deliveries (vaginal delivery with the use of vacuum extractor or cesarean section) were statistically higher in women with MSAF [23]. Fetal and perinatal outcomes were evaluated in this study and no differences were reported regarding the neonatal birth weight, APGAR score at the first minute of life of newborns and activation of the neonatal resuscitation unit.

The strength of this study is in its focus on the overall health of women, analyzing their background without restriction to purely clinical aspects. It provides a comprehensive personalized treatment strategy for the Ethiopian population, which is different from the one needed in the Western population, examining the cultures and traditions of each place with an attentive and respectful approach.

The study is limited by the language barrier, which, particularly during the initial stages of data collection, hindered clear and effective communication. Due to the lack of precise patient addresses, it was necessary to approximate the distance from the hospital to the areas where the mothers reported living.

## 5. Conclusions

Socioeconomic factors of pregnant women in Ethiopia such as family income, weekly workload, living in a rural zone (distance from the hospital), trip duration to reach the hospital and the use of different means of transportation should be considered as risk factors for MSAF. A correct identification of these risks combined with socio-political and cultural action might decrease the prevalence of MSAF in this population and consequently reduce the rate of operative deliveries.

## Figures and Tables

**Table 1:** Comparison of different characteristics in group A (women with meconium-stained amniotic fluid) and group B (women with clear amniotic fluid).

	Group A 65 Women	Group B 100 Women	*p*-Value
Age (years)	26.1 ± 5.1	26.5 ± 5.8	0.69
Multiparity	54%	66%	0.12
Labor duration (hours)	9.1 ± 3.9	9.1 ± 4.9	1
Labor augmentation	10 (15.4%)	15 (15%)	0.94
Premature rupture of membranes (PROM)	6 (9.2%)	11 (11%)	0.71
Oligohydramnios on admission	1 (1.5%)	3 (3%)	N/A
Antenatal care follow-up	37 (56.9%)	61 (61%)	0.6
Distance from hospital (Km)	33.1 ± 54	18.6 ± 27.2	0.02
Distance from hospital > 20 Km	26 (40%)	24(24%)	0.03
Trip duration (minutes)	65.5 ± 75.5	26 ± 33.2	<0.001
Use of vehicles for transportation	41 (63.1%)	95 (95%)	<0.001
Weekly workload > 36 h	63 (96.9%)	70 (70%)	<0.001
Family income (birr)	2149 ± 744	2655 ± 572	<0.001
Number of children at home	1.4 ± 1.8	1.7 ± 1.9	0.34

**Table 2:** Evaluation of maternal and neonatal morbidity in group A (women with meconium-stained amniotic fluid) and group B (women with clear amniotic fluid). * Breech presentations excluded (group B denominator = 97).

	Group A 65 Women	Group B 100 Women	*p*-Value
Temperature elevation during labor	6 (9.2%)	11 (11%)	0.71
Labor dystocia	6 (9.2%)	14 (14%)	0.36
Fetal tachycardia	4 (6.2%)	12 (12%)	0.22
Prolonged decelerations or fetal bradycardia	34 (52.3%)	20 (20%)	<0.001
Operative deliveries (cesarean delivery and vacuum extraction) *	16 (24.6%)	9 (9.3%)	0.008
Neonatal birth weight	3043 ± 401	3040 ± 567	0.97
APGAR score < 7	12 (18.5%)	10 (10%)	0.12
Resuscital care	8 (12.3%)	5 (5%)	0.09

## Data Availability

The data supporting the findings of this study are available from the corresponding author upon reasonable request.

## Abbreviations

The following abbreviations are used in this manuscript:

MSFA	Meconium-stained amniotic fluid
MAS	Meconium aspiration syndrome
AFSOL	Active first stage of labor
LFSOL	Latent first stage of labor
PROM	Premature rupture of membranes
